# Interstitial Lung Disease Associated with Anti-Ku Antibodies: A Case Series of 19 Patients

**DOI:** 10.3390/jcm14010247

**Published:** 2025-01-03

**Authors:** Laure Petitgrand, Kaïs Ahmad, Delphine Gamondès, Rémi Diesler, Nicole Fabien, Laure Gallay, Romain Fort, Julie Traclet, François Lestelle, Roland Chapurlat, Cyrille B. Confavreux, Stéphane Durupt, Ségolène Turquier, Salim Aymeric Si-Mohamed, Frédéric Coutant, Vincent Cottin

**Affiliations:** 1 Department of Respiratory Medicine, National Reference Center for Rare Pulmonary Diseases, Louis Pradel Hospital, Hospices Civils de Lyon, European Reference Network (ERN)-LUNG, 28 Avenue Doyen Lepine, 69677 Lyon, France; laure.petitgrand@live.fr (L.P.); kais.ahmad@chu-lyon.fr (K.A.); remi.diesler@gmail.com (R.D.); julie.traclet@chu-lyon.fr (J.T.); francois.lestelle@chu-lyon.fr (F.L.); 2 Radiology Department, Louis Pradel Hospital, Hospices Civils de Lyon, 69677 Lyon, France; delphine.gamondes@chu-lyon.fr (D.G.); salim.si-mohamed@chu-lyon.fr (S.A.S.-M.); 3 Immunology Department, Lyon-Sud Hospital, Hospices Civils de Lyon, 69149 Pierre Bénite, France; nicole.fabien@chu-lyon.fr (N.F.); frederic.coutant@chu-lyon.fr (F.C.); 4 Immunogenomics and Inflammation Research Team, Faculté Lyon Sud, Claude Bernard University Lyon 1, 69367 Lyon, France; 5 Department of Clinical Immunology, Edouard Herriot Hospital, Hospices Civils de Lyon, 69367 Lyon, France; laure.gallay@chu-lyon.fr; 6 Department of Anesthesia and Intensive Care Unit, Lyon Sud Hospital, Hospices Civils de Lyon, 69149 Pierre Bénite, France; romain.fort@chu-lyon.fr; 7 INSERM UMR 1033, Faculté Lyon Sud, Université Claude Bernard-Lyon 1, Edouard Herriot Hospital, 69367 Lyon, France; roland.chapurlat@chu-lyon.fr (R.C.); cyrille.confavreux@chu-lyon.fr (C.B.C.); 8 Rheumatology Department, Lyon Sud Hospital, Cancer Institute of Hospices Civils de Lyon, 69003 Lyon, France; 9 Department of Internal Medicine, Lyon Sud Hospital, Hospices Civils de Lyon, 69149 Pierre Bénite, France; s.durupt@resamut.fr; 10 Pulmonary Physiology, Louis Pradel Hospital, Hospices Civils de Lyon, 69677 Lyon, France; segolene.turquier@chu-lyon.fr; 11 UMR 754, INRAE, Faculté Lyon Est, Claude Bernard University Lyon 1, 69367 Lyon, France

**Keywords:** autoimmunity, pulmonary fibrosis, connective tissue disease

## Abstract

**Background:** Antibodies against Ku have been described in patients with various connective tissue diseases. The objective of this study was to describe the clinical, functional, and imaging characteristics of interstitial lung disease in patients with anti-Ku antibodies. **Methods**: This single-center, retrospective observational study was conducted at a tertiary referral institution. Patients with positive anti-Ku antibodies and interstitial lung disease identified between 2007 and 2022 were included. Clinical, immunological, functional, and imaging data were systematically reviewed. **Results**: Nineteen patients (ten females) with a mean age of 59 ± 12.6 years were included. The most frequent associated diagnosis was systemic sclerosis (42%), followed by rheumatoid arthritis (26%), Sjögren syndrome, undifferentiated connective tissue disease, and overlap between systemic sclerosis and idiopathic inflammatory myopathy (scleromyositis). Imaging revealed frequent septal and intralobular reticulations and ground-glass opacities, with nonspecific interstitial pneumonia as the predominant pattern (53%). The mean forced vital capacity was 82% ± 26 of the predicted value, and the mean diffusing capacity for carbon monoxide was 55% ± 21. Over the first year of follow-up, the mean annual forced vital capacity decline was 140 mL/year (range: 0–1610 mL/year). The overall survival rate was 82% at 5 years and 67% at 10 years. **Conclusions**: Most patients with interstitial lung disease and anti-Ku antibodies presented with dyspnea, a mild-to-moderate restrictive ventilatory pattern, and reduced diffusing capacity for carbon monoxide. The CT pattern was heterogeneous but was consistent with nonspecific interstitial pneumonia in half of the patients.

## 1. Introduction

Interstitial lung diseases (ILDs) encompass a wide spectrum of conditions with diverse etiologies, clinical presentations, imaging features, and outcomes [1]. ILDs can be classified into disease categories based on the mode of onset, presumed pathophysiology, or management purposes. ILDs can be further divided into known and unknown causes or etiological contexts. Those occurring on the basis of a known underlying disease include connective tissue disease (CTD)-associated ILD, also known as systemic autoimmune rheumatic disease, hypersensitivity pneumonitis, drug-induced ILD, and other ILDs associated with occupational and environmental exposures.

ILD is one of the more severe forms of organ involvement in patients with CTD. It is present in approximately 11% of patients with rheumatoid arthritis, 47% of those with systemic sclerosis (SSc), 41% of those with idiopathic inflammatory myopathy (IIM), 17% of those with primary Sjögren syndrome, 6% with mixed CTD, and 6% with systemic lupus erythematosus (SLE) [2]. In all CTDs, ILD is associated with an excess of morbidity and mortality [3]. The imaging and histopathologic patterns of ILD vary according to the underlying CTD. Nonspecific interstitial pneumonia (NSIP) is the most common pattern in CTD-ILD, particularly in SSc, IIM, and mixed CTD [2].

The diagnostic approach of ILD is complex and best conducted by a multidisciplinary team in specialized centers [4] to integrate clinical, radiological, physiological, biological, and sometimes histological findings. The nature and severity of lung disease may vary according to the CTD category and autoantibody [3,5], as specific serological autoantibodies are often associated with the occurrence and clinical course of ILD in people with CTD [6,7]. The identification of autoantibodies also facilitates the diagnosis and classification of CTDs, especially when ILD precedes extra-respiratory manifestations, as is often the case with IIM [8,9,10]. Autoantibodies found in patients with SSc include antitopoisomerase-1 (anti-Scl-70) antibodies, anti-centromere antibodies, anti-RNA polymerase-3 antibodies, and more rare ones, including anti-Ku antibodies [11]. Numerous autoantibodies may be associated with IIM, e.g., antisynthetase antibodies, myositis-specific antibodies, and myositis-associated autoantibodies, including anti-Ro/SSa, anti-U1RNP 70 kDa, anti-PM/Scl 75 and 100 kDa, and anti-Ku antibodies [12]. Anti-PM/Scl and anti-Ku autoantibodies are frequently associated with IIM overlapping with SSc (scleromyositis) [9,10,13,14].

Anti-Ku antibodies are autoantibodies targeting the Ku protein, which is an 80 and 70 kDa DNA-binding protein involved in the DNA repair pathway for double-strand breaks and preventing telomere shortening [15]. Telomere degradation results in cellular replication arrest and premature cell death. This mechanism has been described in familial pulmonary fibrosis linked to *TERC* and *TERT* gene mutations, among others [16]. Anti-Ku antibodies were first identified in overlap syndromes involving IIM/SSc in 1981 by Mimori et al. [17], but they are also found in other autoimmune diseases such as SSc, SLE, IIM, and mixed CTD [18]. The prevalence of anti-Ku antibodies in each of these conditions varies greatly across studies, ranging from 1% to 16% in SSc, up to 20% in Sjögren syndrome, and as high as 26% in IIM [19], likely due to the heterogeneity of the study population and immunologic detection methods across studies [20,21].

Although previous studies have explored the clinical and systemic phenotype associated with anti-Ku antibodies [18,22,23,24,25,26,27,28,29,30,31,32,33], limited data are available on the specific features and progression of lung involvement in patients with these antibodies. The present study aims to characterize ILD in patients with anti-Ku antibodies, with a focus on clinical, imaging, and functional aspects of lung disease.

## 2. Patients and Methods

### 2.1. Study Design and Eligibility Criteria

This single-center retrospective observational study was conducted at a referral institution (Hospices Civils de Lyon, HCL). The inclusion criteria were the identification of anti-Ku antibodies between 2007 and August 2022, confirmed in the immunology department of the institution, and the presence of ILD on chest CT scans confirmed by a chest radiologist (D.G.). Cases with a positive anti-Ku antibody test between 2007 and 2022 were identified using the HCL centralized immunology laboratory’s computerized records. Patients under 18 years old at data collection (January 2021–August 2022) were excluded.

### 2.2. Antibody Testing

Autoantibody testing was performed by two of the authors (N.F. and F.C.) in the immunology laboratory of the HCL. Anti-Ku antibody identification was performed using the dot immunoassay (Euroimmun, Bussy-St Martin, France), with a positive threshold set at 15 according to the manufacturer’s instruction and derived from patients with autoimmune diseases, excluding myositis, and healthy control subjects. Nuclear antibodies fluorescence patterns were assessed using indirect immunofluorescence on HEp-2 cells (Kallestadt, Biorad, Marnes la Coquette, France) as anti-Ku antibodies typically exhibit a speckled fluorescence pattern, with fluorescence around the chromatin and negative mitoses in the metaphase and telophase. Patients with false positive anti-Ku results, i.e., weak-intensity and/or inconsistent immunofluorescence findings were excluded. In all cases, the clinical diagnosis of the autoimmune disease was established by a consultant rheumatologist or an internal medicine specialist.

### 2.3. Clinical and Radiological Data Collection

Clinical, biological, functional, and radiological data were extracted from electronic medical records. Pulmonary function tests were consistently performed using the same spirometer in each individual. The reference standards used were those of the Global Lung Initiative.

Chest CT scans were analyzed by a chest radiologist with 20 years of experience (D.G.) and a pulmonologist specializing in ILD (K.A., 10 years of experience, from the National Coordinating Reference Center for Rare Pulmonary Diseases). Discrepancies were adjudicated by another thoracic radiologist (S.S.-M.). Collected imaging data included confirmation of ILD presence, ILD features, distribution of the lesions, and the ILD pattern. CT scans were performed with thin sections without contrast injection.

### 2.4. Ethics

In accordance with French regulations, each patient was informed through a notice letter about their right to oppose the collection of their personal data. This study was conducted according to the guidelines of the Declaration of Helsinki and was registered with the CNIL (National Commission on Informatics and Liberties): registration no. 22-1789. The experiment was approved by the Institutional Review Board of HCL and National Commission on Informatics and Liberties (22-1789, on 3 February 2023).

### 2.5. Statistical Analysis

Qualitative variables are reported as frequencies and percentages, and quantitative variables are expressed as the median (interquartile range [IQR]) or means ± standard deviation counts (proportion (%)), whichever was appropriate, along with range. Normality of the data was verified by the Shapiro–Wilk test. The statistical analyses were performed using Excel software (version 2410).

## 3. Results

### 3.1. Study Population

**Figure 1:**
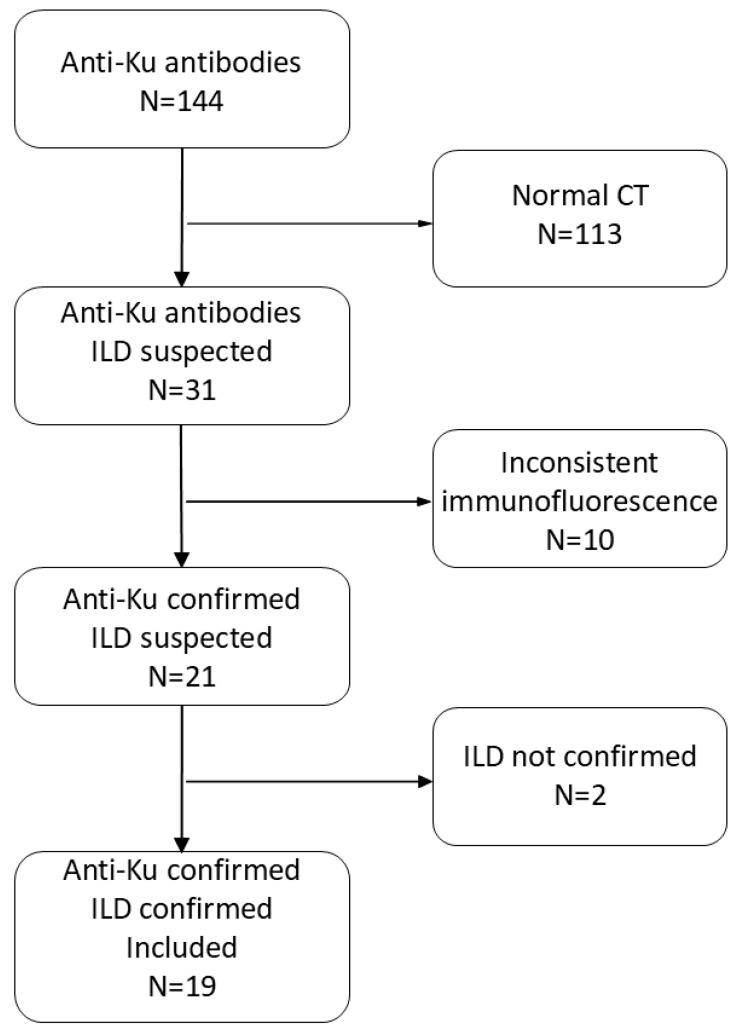
Flowchart of the study. CT: computed tomographyl; ILD: interstitial lung disease.

**Figure 2:**
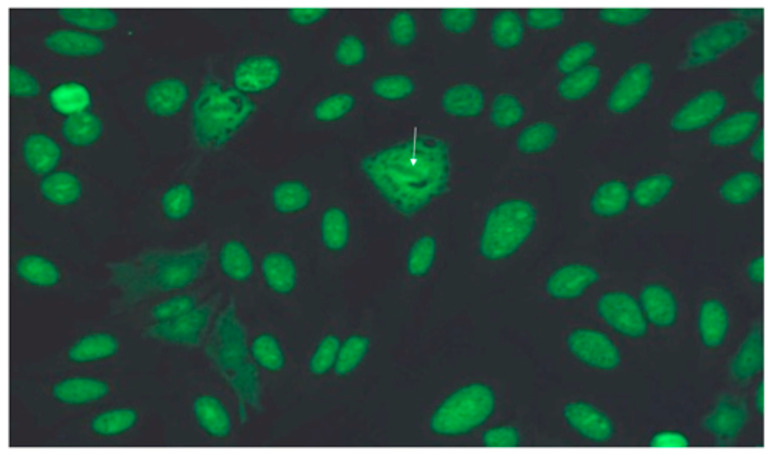
Characteristic speckled nuclear fluorescence pattern of anti-Ku antibodies observed on HEp-2 cells by indirect immunofluorescence. The white arrow highlights the nucleus of a cell in metaphase with fluorescence around the chromatin and negative mitoses.

Among 144 serum samples with anti-Ku antibodies detected using the dot assay, 31 were associated with patients presenting diffuse abnormalities on chest CT scan (Figure 1). Upon further review of the immunology data, 10 cases were considered false positives based on an inconsistent indirect immunofluorescence pattern on HEp-2 cells (Figure 2) or low intensity close to the positivity threshold. However, one patient with an atypical homogeneous fluorescence pattern was maintained in the cohort due to the presence of an anti-chromatin antibody, which may have masked the speckled nuclear pattern typically associated with anti-Ku antibodies. Additionally, two samples exhibited both nuclear and cytoplasmic fluorescence without a clearly identified target.

Two cases were further excluded upon review of the clinical data and chest CT scans because of the absence of definite ILD. In one case, the final diagnosis was asthma with bronchial dilation and micronodules related to an intercurrent infection. The other patient had interstitial abnormalities secondary to SARS-CoV-2 infection, which resolved on follow-up scan.

In total, 19 patients with confirmed anti-Ku antibodies, typical fluorescence pattern, and confirmed ILD on chest CT scan were included in the final cohort.

### 3.2. Patient Characteristics

The sex ratio was balanced, with 53% of the participants being female (n = 10) (Table 1). The mean age at diagnosis of autoimmune disease was 59 years ± 12.6 (range: 26–76 years), while the mean age at diagnosis of ILD was 61 years ± 11.7 (range: 40–82 years). The average time interval between autoimmune disease diagnosis and ILD onset was 2 years (range: 0–15 years). ILD was diagnosed before the onset of autoimmune disease in none of the patients. The mean duration of ILD follow-up was 7 years ± 4.9 (range: 0–15.3 years).

Eight patients were current or former smokers (42%). Moderate occupational or environmental exposure was noted in two patients (11%), but it was not considered causative of ILD. At the time of autoimmune disease diagnosis, most patients presented with dyspnea (79%) with a median modified Medical Research Council score of 2 and crackles on auscultation (53%). Four patients reported coughing (21%), and six patients (32%) reported general decline in health status. One patient exhibited finger clubbing.

### 3.3. Characteristics of Autoimmune Disease

The diagnosis of autoimmune disease is summarized in Table 2. The most frequent diagnosis was SSc (42%), followed by rheumatoid arthritis (26%). Idiopathic inflammatory myopathy was diagnosed in 10% of the cases, and an overlap of SSc and IIM (scleromyositis) was observed in 14% of the cases.

Arthralgia was reported in twelve patients (63%), with only one patient showing radiographic evidence of joint destruction (Table 3). Muscle weakness was present in six patients (32%), including three with muscle atrophy (16%) and two experiencing loss of ambulation (11%). Creatine phosphokinase levels were measured in six patients, with a mean of 681 IU/L ± 420 (range: 233–1177 IU/L). Four patients (21%) exhibited a myogenic pattern on electromyography, and muscle biopsy revealed myositis in two patients (11%).

Scleroderma-related symptoms included Raynaud’s phenomenon in eleven patients (58%), sclerodactyly in eight (42%), limited mouth opening in five (26%), telangiectasia in one (5%), and subcutaneous calcinosis in one (5%). Capillaroscopy, when performed, was positive in seven patients (37%) with findings of giant capillaries, hemorrhage, or capillary rarefaction. Skin ulcers or necrosis was present in three patients (16%) although no patient exhibited vasculitic purpura. Ocular or oral dryness was noted in four patients (26%), with positive salivary gland biopsy findings in four (21%). Schirmer tests were negative in the entire cohort.

Seven patients (37%) had gastroesophageal reflux, and three reported epigastric pain (16%). Esophageal dilation and dysphagia were documented in five patients (26%), and dysphagia in three (16%). Peripheral neuropathy of various etiologies was noted in five patients (26%). The central nervous system was not involved. Pericarditis associated with scleroderma was identified in two patients (11%).

### 3.4. Immunology

The mean intensity of anti-Ku antibodies was 156 UFR ± 49 (range: 84–223 UFR). Antinuclear antibodies were found in all cases, with titers of 640 in two patients, 1280 in sixteen patients, and 160 in one patient. The typical speckled pattern of antinuclear antibodies on HEp-2 cells was detected in eighteen cases (95%), while one patient exhibited a homogeneous immunofluorescence pattern. Indeed, the presence of anti-chromatin antibodies may have masked the typical pattern associated with anti-Ku antibodies. Thirteen patients (70%) had additional antibodies identified (Table 2). Patients with isolated anti-Ku antibodies or additional antibodies exhibited similar characteristics.

### 3.5. Pulmonary Function Tests

The pulmonary function tests at baseline are presented in Table 4. At diagnosis, the mean forced vital capacity (FVC) was 82% of the predicted value, the mean total lung capacity was 77% of the predicted value, and the mean diffusing capacity for carbon monoxide (DLCO) was 55% ± 21. Almost the entire cohort (n = 17; 89%) exhibited diffusion impairment, with decreased DLCO and/or KCO below 70%, and half of the patients (n = 10; 53%) showed a restrictive ventilatory pattern. Only one patient had an obstructive ventilatory defect associated with COPD. Two patients were receiving supplemental oxygen therapy.

Follow-up assessments were conducted annually. The average annual decline in FVC was 140 mL/year (range: 0–1610 mL/year), and the mean annual decrease in DLCO was 0.97%/year (range: 0–4.9%/year).

### 3.6. Imaging Findings

The most common imaging findings were septal reticulations, intralobular reticulations, and ground-glass opacities in most patients (Table 5). Traction bronchiectasis and bronchiolectasis were present in approximately half of the patients (53%). Honeycombing was observed in only three patients (16%). The typical subpleural sparing of the NSIP pattern was observed in only two patients (11%).

**Figure 3:**
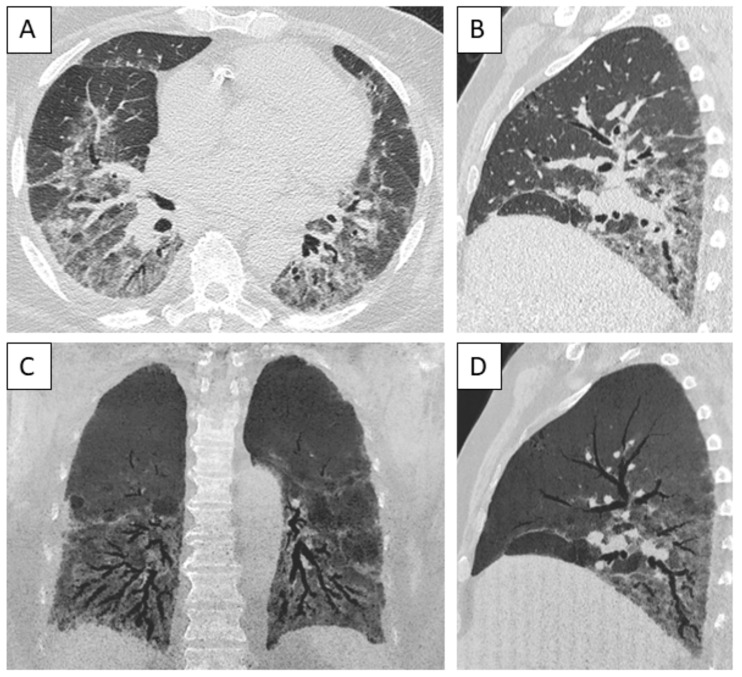
Computed tomography of a male patient with anti-Ku antibodies, demonstrating a pattern of fibrotic nonspecific interstitial pneumonia. (**A**) Axial view of lower part of the lungs, demonstrating diffuse distribution of ground-glass opacities, reticulation, bronchiectasis, and bronchiolectasis. (**B**) Sagittal view showing apicobasal distribution. (**C**) Coronal view, with minimal intensity projection of 13 mm. (**D**) Sagittal view, with minimal intensity projection of 13 mm.

**Figure 4:**
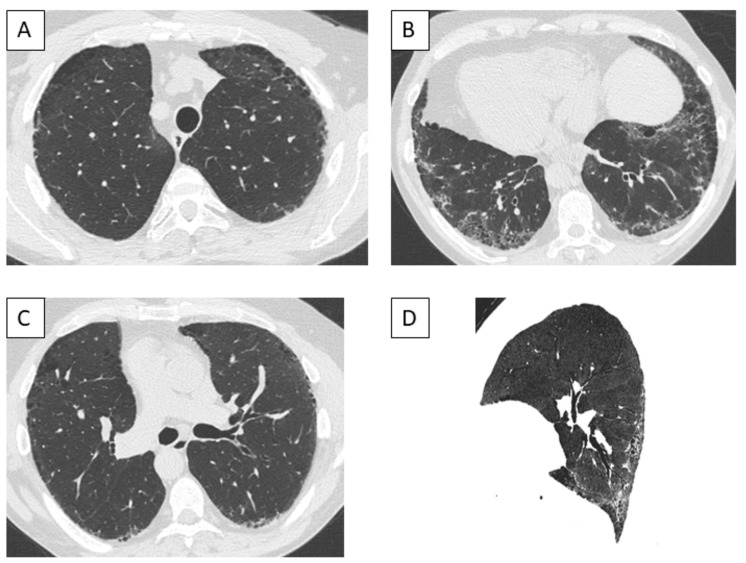
Computed tomography of a male patient with anti-Ku antibodies, demonstrating a pattern of fibrotic nonspecific interstitial pneumonia, apicobasal distribution of ground-glass opacities and reticulation with subpleural sparing, bronchiectasis, and bronchiolectasis, associated with paraseptal emphysema. (**A**) Upper part of the lungs, axial view. (**B**) Middle part of the lungs, axial view. (**C**) Lower part of the lungs, axial view. (**D**) Sagittal view, with a minimal intensity projection of 5 mm.

The most frequent radiological pattern was nonspecific interstitial pneumonia (NSIP), identified in 10 cases (53%); two additional cases had a pattern of NSIP/organizing pneumonia overlap (Figure 3 and Figure 4). No significant difference was observed in the distribution of patterns according to the underlying diagnosis of autoimmune disease.

The extent of ILD comprised 0–25% of the total lung volume in 10 patients (53%), 25–50% of the lung volume in 7 (37%), and >50% of the lung volume in 2 patients (11%). The distribution was predominantly peripheral in 14 cases (74%), and most commonly affected the lower lobes in 9 cases (47%).

To assess the progression of interstitial lung disease (ILD), the initial diagnostic CT scan was compared with the most recent CT scan, with an average follow-up duration of 7 years. A significant majority of patients (n = 13; 68%) showed worsening CT lesions, whereas two patients (11%) had stable disease, and one patient experienced improvement, which was attributed to the initiation of oral glucocorticoid therapy. Three patients did not have a follow-up CT scan available for comparison.

### 3.7. Bronchoalveolar Lavage

Six patients (32%) underwent bronchoscopy with bronchoalveolar lavage at ILD diagnosis. The bronchoalveolar lavage profile was predominantly neutrophilic in five patients (83%) and mixed neutrophilic and lymphocytic in one patient. None of the patients underwent biopsies, whether endoscopic or surgical.

### 3.8. Treatment and Outcome

During the follow-up, treatments administered to the patients included oral glucocorticoids in seventeen patients (89%), mycophenolate mofetil in seven patients (37%), methotrexate in six patients (32%), azathioprine in six patients (32%), hydroxychloroquine in five patients (26%), intravenous pulse cyclophosphamide in four patients (21%), intravenous immunoglobulins in two patients (11%), and rituximab, abatacept, adalimumab, and tocilizumab in one patient each. In total, nearly all patients (89%) received glucocorticoids for more than one month, and 15 patients (79%) were treated with at least one immunosuppressant during follow-up.

Control of both systemic disease and ILD was achieved with glucocorticoids alone in three patients (16%), another immunosuppressive drug alone in four patients (21%), and a combination of glucocorticoids and other immunosuppressants in three patients (16%). Three patients remained clinically stable without specific treatment (16%). Four patients (21%) showed uncontrolled disease progression despite immunosuppressant and/or glucocorticoid treatment, requiring intensified immunosuppressive therapy, and two of these patients subsequently died. Of note, due to the large time span of this retrospective study, none of the patients received antifibrotic therapy. Two patients were recently diagnosed and have not yet undergone treatment reassessment.

Four patients developed group 3 pulmonary hypertension, including three with SSc and one with IIM. Two patients experienced severe respiratory involvement with hypoxemia, necessitating long-term supplemental oxygen therapy. No patient in this cohort underwent lung transplantation.

**Figure 5:**
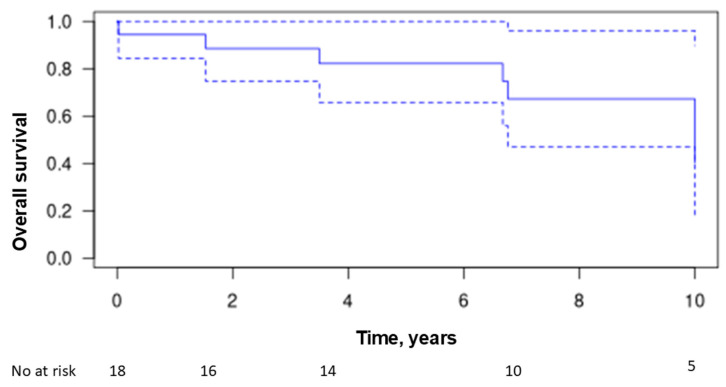
Kaplan–Meier estimates of overall survival. The solid line represents the estimate of overall survival. The dotted lines represent the 95% confidence interval.

Over the follow-up period, seven patients died (37%), with an average time from diagnosis to death of 6.7 years ± 5.5 (range 0–15.3 years). The causes of death were acute respiratory distress in three patients (16%), septic shock in two patients (11%), and macrophage activation syndrome related to lymphoma in one patient. The cause of death was unreported in one patient. The overall survival rate was 82% at 5 years and 67% at 10 years (Figure 5). No patients were lost to follow-up.

## 4. Discussion

In this case series, we described the characteristics of ILD associated with the presence of anti-Ku antibodies. In all cases, ILD onset was chronic, occurring at the onset of the autoimmune disease or shortly thereafter during follow-up. The mean delay between the diagnosis of autoimmune disease and ILD was 2 years. This disease course is consistent with previous series, with SSc and rheumatoid arthritis being the most frequent diagnoses [11,34], with onset of lung involvement within the first few years of the disease course of systemic autoimmune disease. ILD preceded systemic disease in none of the patients.

Clinical respiratory manifestations were nonspecific. Lung function tests were consistent with mild-to-moderate severity in the majority of the cases. The imaging pattern of ILD was that of NSIP in half of the cases, which is the most frequent pattern observed in both SSc and IIM [2]; in two cases, a CT pattern of NSIP/organizing pneumonia overlap was seen, as frequently seen in patients with IIM and especially antisynthetase syndrome [10,35,36]. In five cases (26%), ILD was fibrotic on the chest CT scan, but the pattern was indeterminate for usual interstitial pneumonia (UIP); only two cases had a pattern of probable UIP, and no definite UIP pattern was seen, consistent with SSc being the most frequent diagnostic category in this series [2,11,37].

The most common extra-respiratory symptoms observed in our study were arthralgia (62%) and Raynaud’s phenomenon (58%), similar to a previous case series of patients with anti-Ku antibodies [26,28]. Patients with ILD and anti-Ku antibodies were diagnosed with a spectrum of autoimmune diseases based on rheumatologic evaluation. SSc was the most frequently diagnosis in our study, but other conditions such as rheumatoid arthritis, Sjögren syndrome, undifferentiated CTD, and IIM were also diagnosed. In a study by Rigolet et al. [28], conducted in a referral center for internal medicine, IIM (37% of cases) was more frequent than SSc (23%), and SLE was observed in 23% of cases. ILD was present in only 11/34 patients (37%) [28]. In a study by Spielmann et al. [26] from a tertiary rheumatology department, the predominant diagnosis associated with anti-Ku antibodies was Sjögren syndrome (36%), followed by IIM (24%), overlapping CTD (26%), and SLE (19%), whereas only 5% of their population consisted of SSc. These findings illustrate that anti-Ku antibodies may be associated with a variety of autoimmune diseases, with the frequency distribution likely reflecting patient recruitment patterns across centers. Cases of anti-Ku antibodies with a diagnosis of SSc or IIM seem to be the most frequently associated with ILD. Interestingly, Spielmann et al. identified two subsets of patients with anti-Ku antibodies [26]; in our series, none corresponded to the subset associated with antibodies against double-stranded DNA and a risk of glomerulonephritis, and most if not all of our cases corresponded to the other subset described by Spielmann et al. [26], with an elevated serum level of creatine phosphokinase and an associated risk of ILD. Notably, all six patients tested in our cohort had elevated serum creatine phosphokinase levels.

Anti-Ku antibodies are very rarely identified. As a benchmark, during the period from 2012 to 2022, 23 patients with dermatomyositis and anti-MDA5 antibodies were identified in our center, among whom 18 (78%) had ILD.

Seventy percent of the patients in the current series had another antibody identified in addition to the anti-Ku antibody. This finding is consistent with previous studies [26,28]. Anti-Ku antibodies were associated with other antibodies in 77% of cases in the study by Rigolet et al. [28], primarily rheumatoid factor and anti-SSA/SSB. Anti-Ku antibodies were similarly associated with rheumatoid factor and anti-SSA antibodies in 31% of cases in the study by Spielmann et al. [26]. This high rate of multiple autoantibody positivity underscores the complex autoimmune background often seen in patients positive for anti-Ku antibodies.

Interestingly, of the 29 patients initially identified as anti-Ku-positive, 10 were excluded because of inconsistent indirect immunofluorescence patterns for typical anti-Ku antinuclear antibodies or because of the low intensity identified in the dot immunoassay close to the positivity threshold. Autoantibody testing using the dot immunoassay is commonly performed when systemic autoimmune disease is suspected, and anti-Ku antibodies may be included in the myositis panel. However, it is essential to confirm that patients who are positive for anti-Ku antibodies truly have anti-Ku antibodies. Indeed, it has been already demonstrated that for the dot immunoassay, false positive results are frequent for anti-Ku antibodies which generally show a low intensity [38]. The confirmation on Hep-2 cells is also essential. When a specific antibody test is inconsistent with the antinuclear pattern, a false positive test result may be considered [39,40]. Finding a specific HEp-2 immunofluorescence pattern confers specificity. This added value of immunofluorescence pattern compared to the dot immunoassay has been already described for other autoantibodies such as anti-SRP antibodies [41]. Furthermore, the reactivity for Ku can be due to the presence of antibodies to dsDNA or DNA-binding proteins that are complexed with dsDNA/dsDNA-binding proteins in the serum of patients that then secondarily bind to Ku or dsDNA/dsDNA-binding proteins in an immunoassay [42].

Although the sample size limited an in-depth analysis of disease evolution, our findings on lung function are consistent with progressive ILD patterns in scleroderma observed in larger cohorts, such as the EUSTAR registry [43]. Over the first year of follow-up, we documented various patterns of disease evolution, with lung function improvement (defined as an FVC absolute increase of 5% or more) in five patients (36%), stable disease (FVC variation less than 5%) in six patients (43%), moderate decline (FVC decrease of 5–10%) in two patients (14%), and significant deterioration (FVC decrease > 10%) in one patient (7%). Overall, the mean decline in FVC (140 mL/year) was slightly greater than that observed in the placebo arm of the Senscis trial (93.3 mL/year) that enrolled patients with SSc and ILD involving at least 10% of the lung volume [44].

The management of ILD in our patients was at the discretion of the treating physicians, who followed the current practices. Given the wide treatment period covered by the study and the absence of guidelines until very recently for CTD-ILD [45,46], the treatments received by the patients were extremely heterogeneous. Nevertheless, glucocorticoids and immunosuppressants, including mycophenolate mofetil and cyclophosphamide, are frequently used as first-line therapy for patients with SSc. Alternative immunosuppressants, such as rituximab and tocilizumab, are now being considered in subsets of patients with SSc [47,48]. Methotrexate use was relatively frequent in our cohort, likely due to the high prevalence of arthralgia in our population and not solely in patients with rheumatoid arthritis. None of the patients received antifibrotic therapy in this cohort because of the study period. The management of ILD associated with anti-Ku antibodies needs to be individualized and discussed among multidisciplinary teams [49].

The limitations of this study include its retrospective design and the inherent risk of missing data. Nevertheless, most patients were consistently monitored in a pneumology department specialized in ILD and had nearly complete data for pulmonary function tests and chest CT scans. Importantly, no patient was lost to follow-up. The single-center nature of our study may be seen as a limitation; however, it facilitated the thorough identification of anti-Ku-positive patients through centralized testing within our institution (HCL), which encompasses four large public hospitals serving a large catchment area. Although the sample size was relatively small, anti-Ku antibody-associated ILD is rare and even nationally, the affected population remains limited. Patients were followed up at a national reference center for ILD and other rare lung diseases, ensuring accurate ILD diagnosis and phenotyping. Another limitation was the lack of histopathologic confirmation of ILD, as lung biopsy indications for ILD diagnosis have become increasingly restricted because of the minimal impact on management in the setting of autoimmune disease and increasing attention to morbidity and mortality risks. In this relatively small cohort, the pattern of ILD progression could not be assessed according to current standards [46,47]; further studies are warranted to assess the proportion of patients with ILD and anti-Ku antibodies who develop progressive pulmonary fibrosis and may benefit from antifibrotic therapy.

## 5. Conclusions

In conclusion, in this series of 19 well-characterized patients with ILD and anti-Ku antibodies, we found that the majority of the patients had dyspnea, mild-to-moderate restrictive ventilatory pattern, and reduced DLCO. The CT pattern was heterogeneous but consistent with NSIP in half of the patients.

## Figures and Tables

**Table 1:** Main individual characteristics of 19 patients with ILD and anti-Ku antibodies.

	Sex	Age	Tobacco Smoking	CTD	ANA Titer	ANA Pattern	FVC, %	DLCO, %	CT Pattern	Follow-Up, Yrs	Death
1	M	62	1	IIM	1280	Speckled	96	52	Indeterm. UIP	1.1	N
2	M	68	1	SSc	160	Speckled	48	28	NSIP	0.0	N
3	F	61	0	SM, RA, SjS	1280	Speckled	50	N/A	Indeterm. UIP	6.8	Y
4	F	61	1	SSc, RA, SjS	1280	Speckled	106	51	Indeterm. UIP	11.1	N
5	F	65	0	SSc	1280	Speckled	81	23	NSIP	11.6	Y
6	F	40	1	SSc	1280	Speckled	96	75	Indeterm. UIP	9.6	N
7	M	76	0	UCTD, PMR	1280	Speckled	61	N/A	NSIP/OP	6.7	Y
8	F	69	0	UCTD	1600	Speckled	81	54	NSIP	3.5	Y
9	M	59	1	IIM	1280	Speckled	76	70	Indeterm. UIP	1.7	N
10	M	73	0	UCTD	1280	Speckled	86	75	Probable UIP	9.9	N
11	F	26	0	RA	1280	Speckled	75	77	Probable UIP	15.2	N
12	F	55	0	UCTD, SjS	1280	Speckled	55	70	NSIP/OP	11.8	N
13	M	61	0	SSc	1280	Speckled	33	24	NSIP	0.0	Y
14	F	66	0	SM	1600	Speckled	87	35	NSIP	15.3	Y
15	M	45	1	SSc	1280	Speckled	99	58	NSIP	5.4	N
16	M	75	1	SM	1280	Speckled	90	26	NSIP	1.5	Y
17	M	50	0	SSc, RA	1280	Speckled	119	81	NSIP	7.6	N
18	F	66	0	RA, SjS	1280	Homogeneous	133	82	NSIP	9.2	N
19	F	53	1	SSc	1280	Speckled	79	47	NSIP	4.1	N

F: female; Indeterm. UIP: indeterminate for usual interstitial pneumonia; IIM: idiopathic inflammatory myopathy; M: male; NSIP: nonspecific interstitial pneumonia; OP: organizing pneumonia; PMR: polymyalgia rheumatica; RA: rheumatoid arthritis; SjS: Sjögren syndrome; SM: scleromyositis; SSc: systemic sclerosis; UCTD: undifferentiated connective tissue disease.

**Table 2:** Diagnosis of autoimmune disease and autoantibodies in 19 patients with ILD and anti-KU antibodies. Eight patients (42%) were classified into more than one category due to various overlap syndromes.

**Clinical Autoimmune Disease Diagnosis**	**N (%)**
Systemic sclerosis	8 (42)
Rheumatoid arthritis	5 (26)
Sjögren syndrome	4 (21)
Undifferentiated connective tissue disease	4 (21)
Scleromyositis	3 (14)
Idiopathic inflammatory myopathy	2 (10)
Polymyalgia rheumatica	1 (5)
**Autoantibodies**	**N (%)**
Anti-Ku	19 (100)
Antinuclear antibodies	19 (100)
Typical speckled pattern	18 (95)
Homogeneous pattern	1 (5)
Other autoantibodies	
Rheumatoid factor	4 (21)
Anti-SSA	2 (11)
Anti-TRIM21	2 (11)
Anti-PMScl	1 (5)
Anti-RNA-polymerase-3	1 (5)
Anti-JO1	1 (5)
Anti-EJ	1 (5)
Anti-PL7	1 (5)
Anti-fibrillarin	1 (5)
Anti-chromatin	1 (5)
Anti-NOR90	1 (5)
Anti-cyclic citrullinated peptide	1 (5)

**Table 3:** Clinical characteristics at ILD diagnosis in the 19 patients. ILD: interstitial lung disease.

	Mean ± SD or N (%)
Age at ILD diagnosis	61 ± 11.7
Sex, female	10 (53%)
**Medical history**	
Hypertension	5 (26%)
Atrial fibrillation	5 (26%)
Venous thromboembolism	4 (21%)
Cancer	4 (21%)
Hypothyroidism	3 (16%)
Type 2 diabetes	2 (11%)
Ischemic cardiomyopathy	3 (16%)
Chronic renal failure	1 (5%)
Obstructive sleep apnea syndrome	1 (5%)
**Respiratory manifestations**	
Shortness of breath	6 (32%)
Cough	4 (21%)
Crackles	10 (53%)
Finger clubbing	1 (5%)
**Other organ manifestations**	
Arthralgia	12 (63%)
Myalgia	6 (32%)
Muscle weakness	6 (32%)
Skin ulcers or necrosis	3 (16%)
Calcinosis	1 (5%)
Raynaud’s phenomenon	11 (58%)
Sclerodactyly	8 (42%)
Mouth-opening limitation	5 (26%)
Telangiectasiae	1 (5%)
Gastroesophageal reflux	7 (37%)
Epigastric pain	3 (16%)
Dysphagia	3 (16%)
Pericarditis	2 (11%)
Sicca syndrome	4 (21%)
Peripheral neuropathy	5 (26%)

**Table 4:** Pulmonary function tests of 19 patients with ILD and anti-Ku antibodies.

Parameter	Mean ± SD
Forced vital capacity (L)	3.04 ± 0.82
Forced vital capacity, % of predicted	82 ± 26
Forced expiratory volume in 1 s	2.22 L ± 0.89
Forced expiratory volume in 1 s, % of predicted	80 ± 26
Forced expiratory volume in 1 s/forced vital capacity, absolute	0.80 ± 0.096
Total lung capacity (L)	4.27 L ± 1.59
Total lung capacity, % of predicted	77 ± 23
Diffusion capacity of carbon monoxide, % of predicted	55 ± 21
Diffusion coefficient, % of predicted	80 ± 22

**Table 5:** CT findings of 19 patients with ILD and anti-Ku antibodies. CT: computed tomography.

**CT Features**	**n (%)**
Reticulation	16 (84)
Intralobular reticulation	14 (74)
Ground-glass opacities	9 (68)
Traction bronchiectasis	10 (53)
Traction bronchiolectasis	10 (53)
Honeycombing	3 (16)
Cysts	3 (16)
Emphysema	3 (16)
Micronodules	1 (5)
Consolidations	1 (5)
Mediastinal lymphadenopathy	7 (37)
Subpleural sparing	2 (11)
**CT patterns**	
Nonspecific interstitial pneumonia	10 (53)
Nonspecific interstitial pneumonia/organizing pneumonia	2 (11)
Indeterminate for usual interstitial pneumonia	5 (26)
Probable usual interstitial pneumonia	2 (11)
Definite usual interstitial pneumonia	0 (0)

## Data Availability

The data supporting the reported results can be obtained from the first author upon reasonable request.
